# Supplementary material for: GEMC1 and MCIDAS interactions with SWI/SNF complexes regulate the multiciliated cell-specific transcriptional program
Source: Cell Death Dis. 2023 Mar 17;14(3):201. doi: 10.1038/s41419-023-05720-4 (PMC10023806; doi:10.1038/s41419-023-05720-4)
Supplement: Supplementary file 1 — Supplementary Table S1 and Figures S1-S7 [file 41419_2023_5720_MOESM1_ESM.pdf]

Lewis, et al. CDD-22-3837

GEMC1 and MCIDAS interactions with SWI/SNF complexes regulate the multiciliated cell-specific transcriptional program

Supplementary Material

**Antigen**	**Vendor**	**Cat#**	**Species**	**Dilution**	**Application**
FLAG	Sigma	F1804	Mouse	1:200	IF, PLA
FLAG	Sigma	F7425	Rabbit	1:200	Western
ARID1A	Sigma	HPA005456	Rabbit	1:100	Western
BRD9	Bethyl	A303-781A	Rabbit	1:100	Western, PLA
BAF57	Bethyl	A300-810A	Rabbit	1:100	Western
BAF155	Abcam	ab72503	Rabbit	1:100	Western
Actin	Sigma	A4700	Mouse	1:10,000	Western
HA	Santa Cruz	sc-7392	Mouse	1:200	IP
HA	Santa Cruz	sc-805	Rabbit	1:4000	Western
Myc	Santa Cruz	sc-789	Mouse	1:4000	Western
Centrin	Sigma	04-1624	Mouse	1:200-1:2000	IF
Acetylated tubulin	Sigma	T6793	Mouse	1:500-1:2000	IF
DEUP1	Kind gift from Andrew Holland	-	Rabbit	1:500	IF
Deup1	Sigma	HPA010986	Rabbit	1:400	IF
ZO-1	Santa Cruz	R40.76	Rat	1:1000	IF
p73	Abcam	ab40658,	Rabbit	1:200	IF
FOXJ1	Kind gift from S. Brody	-	Rabbit	1:400	IF
**Secondaries**	**Vendor**	**Cat#**	**Species**	**Dilution**	**Application**
anti-Mouse IgG (H+L) Alexa Fluor 680	Thermo Fisher	A-21057	Goat	1:500	IF
anti-rabbit IgG (H+L) Alexa Fluor 680	Thermo Fisher	A-21076	Goat	1:500	IF
anti-Mouse IgG2b, Alexa Fluor-488	Thermo Fisher	A-21141	Goat	1:500	IF

anti-Mouse IgG2a, Alexa Fluor-568	Thermo Fisher	A-21134	Goat	1:500	IF
anti-mouse HRP conjugate	Promega	W4028	Goat	1:15000	IF
anti-rabbit HRP conjugate	Promega	W4018	Goat	1:15000	IF
IRDye® 680LT Goat anti-Rabbit IgG Secondary Antibody	Li-COR	925-68021	Goat	1:15000	WB
IRDye® 680LT Goat anti-Mouse IgG Secondary Antibody	Li-COR	926-68020	Goat	1:15000	WB
IRDye® 800CW Goat anti-Mouse IgG Secondary Antibody	Li-COR	926-32210	Goat	1:15000	WB
IRDye 800CW streptavidin	Li-COR	926-32230	Goat	1:10000	WB

Supplementary Table S1: Antibodies used in this study. Applications are indicated in the far right column.

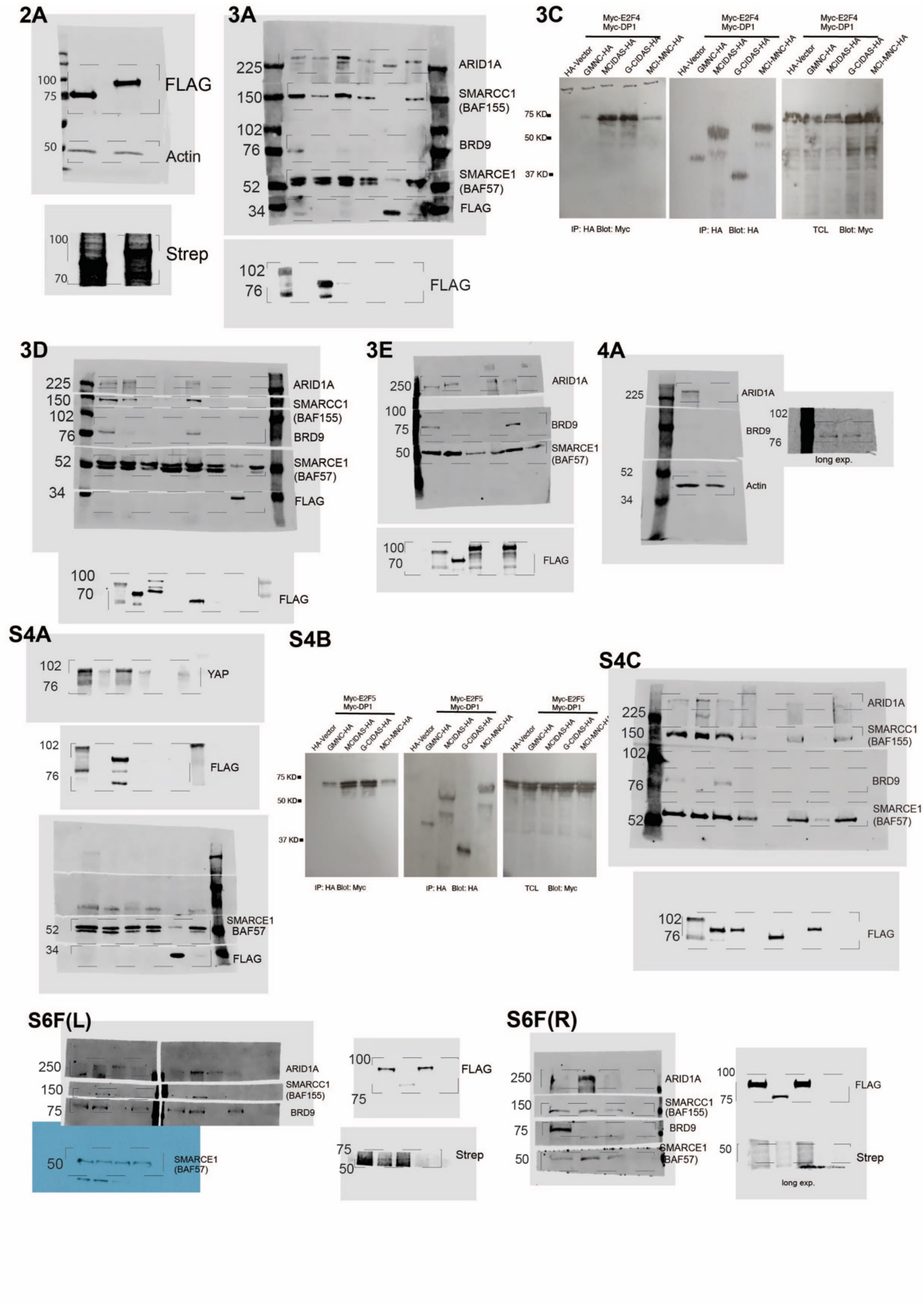

Supplementary Figure S1: All uncropped western blots. Corresponding figures are indicated for each.

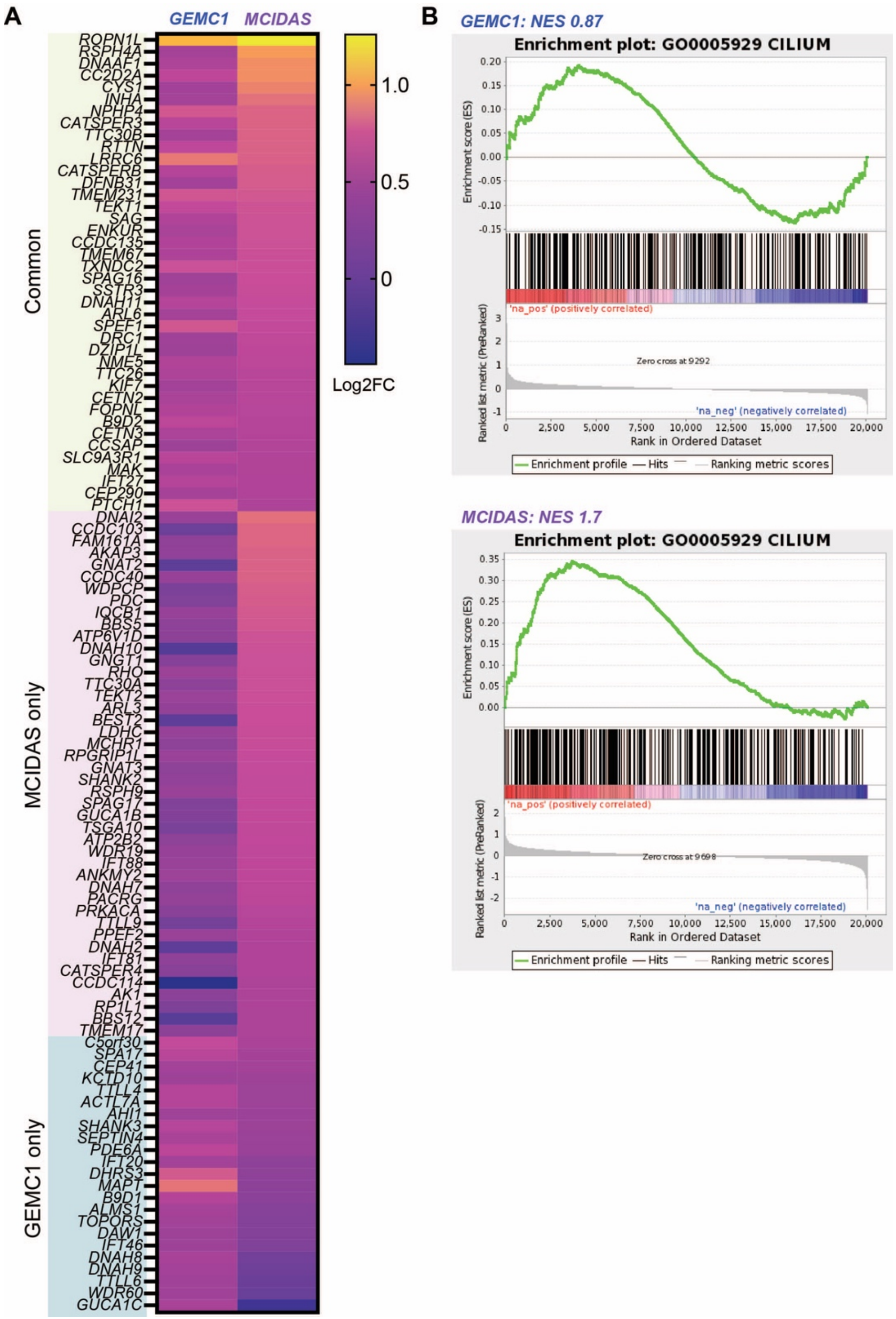

Supplementary Figure S2: Heatmap of genes in the Cilium gene ontology category. **A.** Heatmap of differentially expressed genes leading to the enrichment of the cilium gene ontology category. Genes contributing to the score of both GEMC1 and MCIDAS (common) or either individual gene are indicated. **B.** Geneset enrichment analysis (GSEA) plots with the Nominal Enrichment Score (NES) for the indicated gene are shown. Gene expression data for all genes can be found in Supplementary Table S2 and additional GSEA data in Supplementary Table S3.

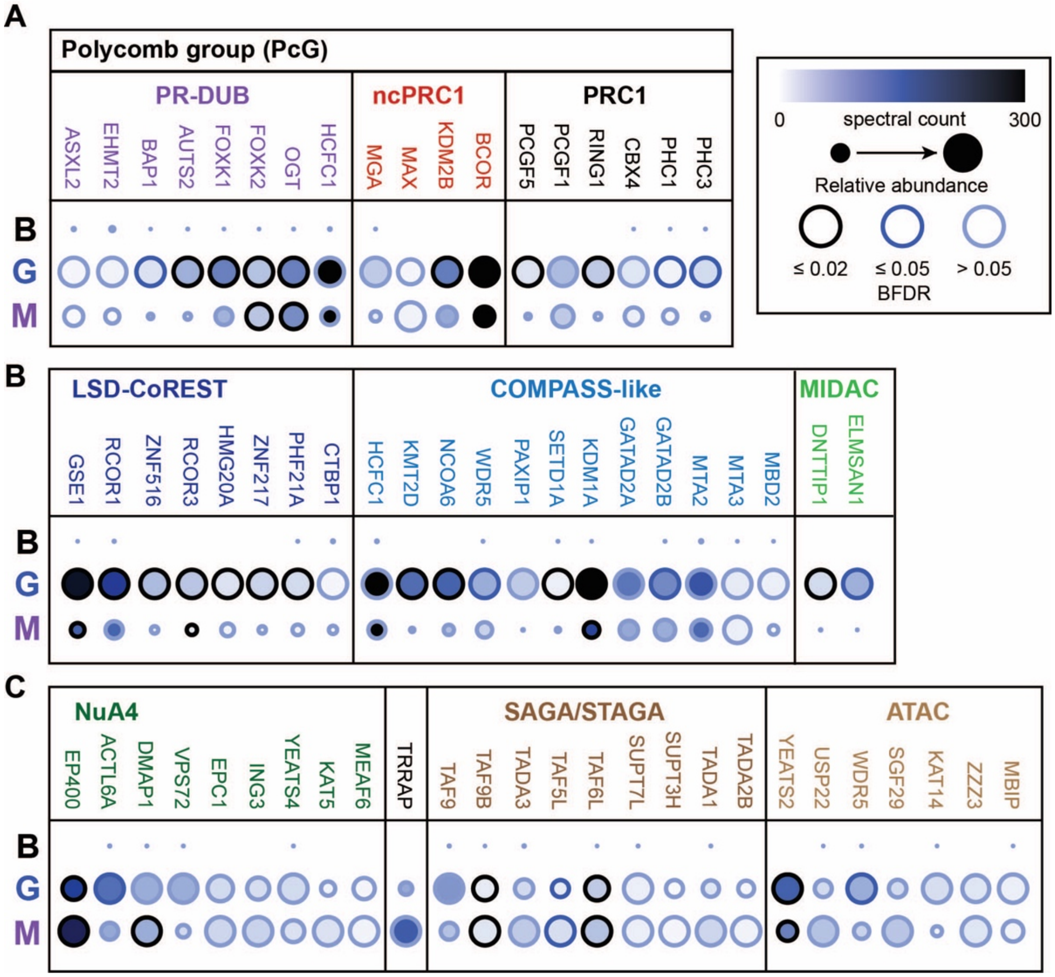

Supplementary Figure S3: Proximal transcription factor complexes of GEMC1 and MCIDAS. A. Dot plot depicting relative abundance and spectral counts for selected Polycomb group (PcG) proteins grouped by known complexes. Key applies to all panels. **B.** Dot plot depicting relative abundance and spectral counts for selected complexes enriched with GEMC1 compared to MCIDAS. **C.** Dot plots of transcriptional complexes associated with TRRAP. B=BirA*, G=GEMC1 and M=MCIDAS for all panels.

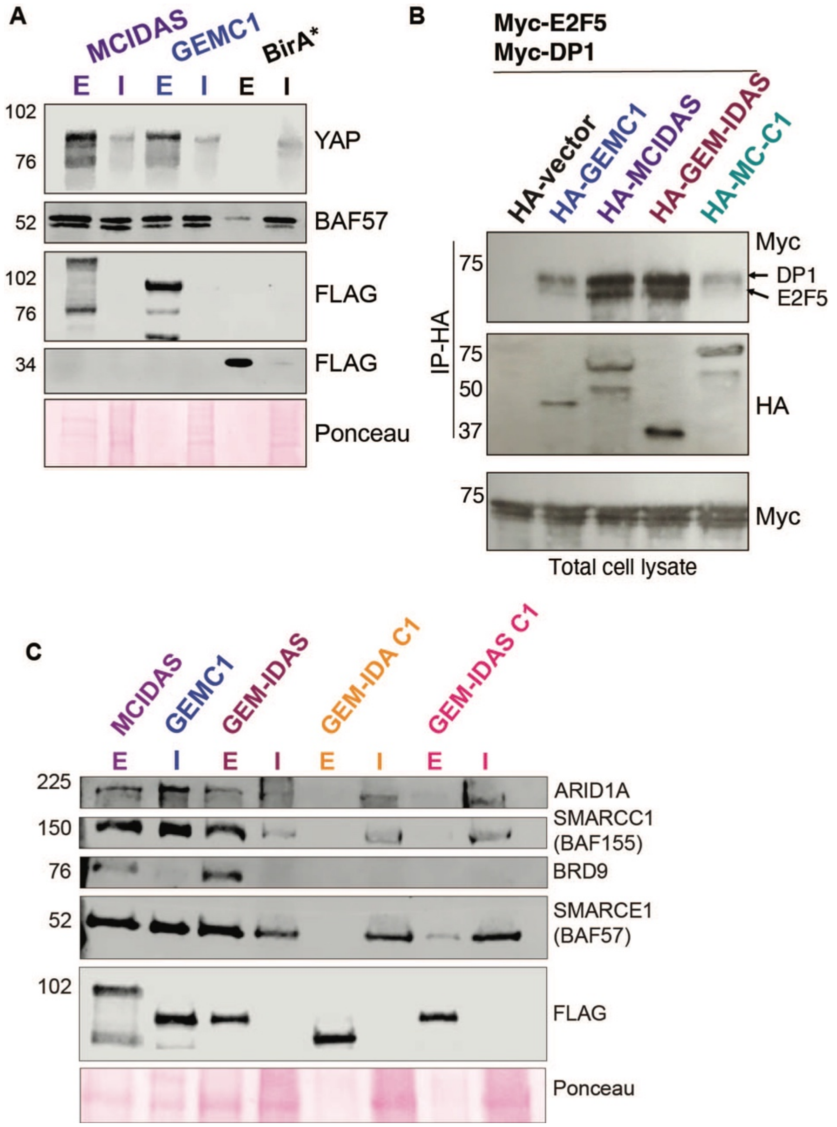

Supplementary Figure S4: Validation of BioID-MS interactions. A. BioID-AP western blots for YAP1, BAF57 and baits (FLAG). Input (I) and streptavidin Eluate (E) are indicated for each gene and ponceau shows similar input loading and transfer efficiencies. **B.** Co-immunoprecipitation experiments demonstrate enhanced E2F5-DP1 interactions with the MCIDAS C-terminus. HEK293T cells were transfected with HA-tagged vector, GEMC1, MCIDAS, GEM-IDAS and MC-C1, Myc-tagged DP1 and Myc-E2F5. Lysates were immunoprecipitated with anti-HA antibodies and westerns carried out for Myc and HA following transfer to PVDF. Total lysates are shown blotted for Myc. Data presented is representative of 2 biological replicates **C.** BioID-AP westerns blots for ARID1A, BRD9 and core SWI/SNF components following expression of indicated hybrid proteins (see Figure 3B for schematic). Ponceau staining shown for loading and transfer control.

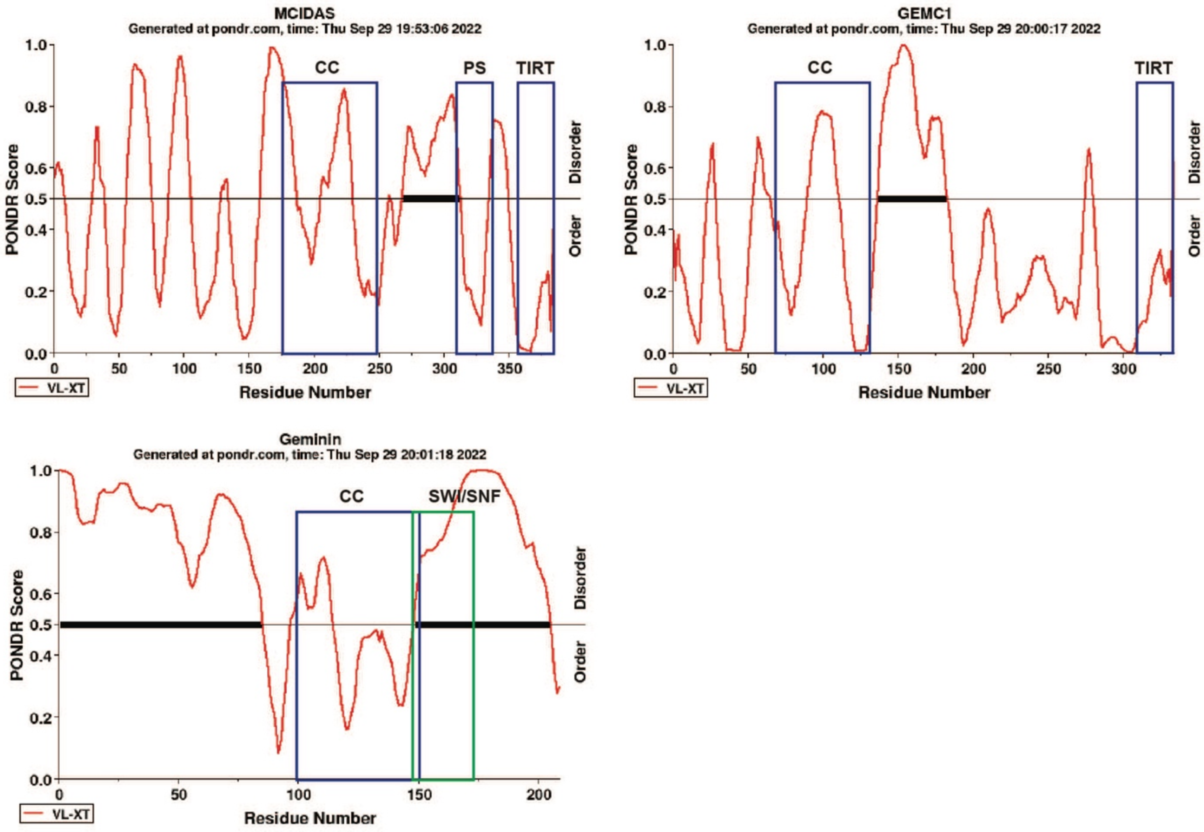

Supplementary Figure S5: Predicted regions of disorder in Geminin, GEMC1 and MCIDAS. Disorder of the indicated proteins predicted by PONDR. The coiled coil (CC), TIRT domains, possibly structured domain of MCIDAS (PS; see Figure 3) and SWI/SNF interacting region of Geminin are indicated.

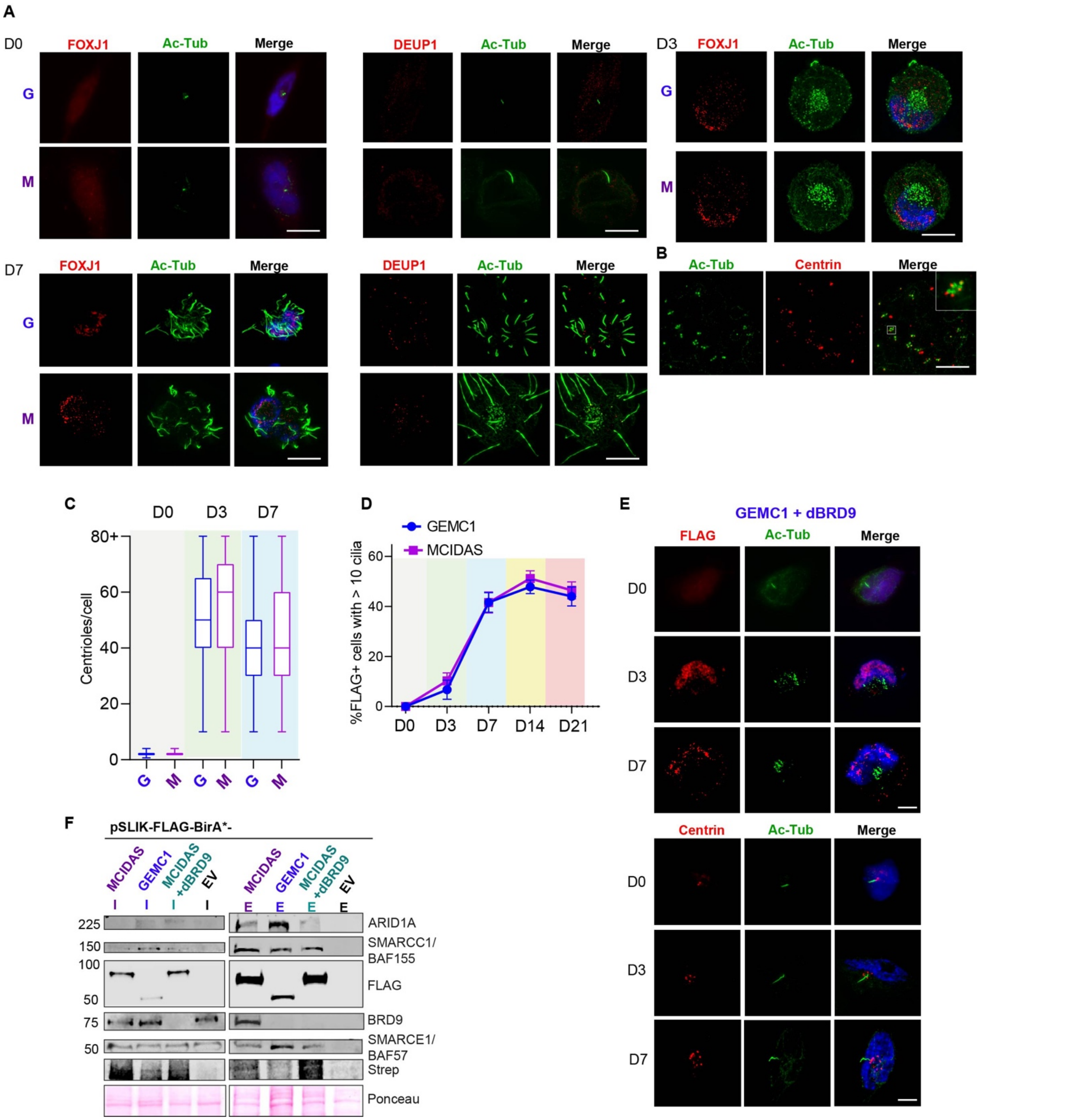

Supplementary Figure S6: Analysis of ciliogenesis in GEMC1 or MCIDAS expressing glioma cells. A. Immunofluorescence of expansion microscopy samples using the indicated markers in cells expressing either G=pSLIK-FLAG-BirA*-GEMC1 or M=pSLIK-FLAG-BirA*-MCIDAS. Images are taken at three different time points: Day 0, Day 3 and Day 7 using 3D-SIM. **B.** Immunofluorescence of expansion microscopy samples using the indicated markers. **C.** Quantification of centriole numbers in the indicated samples using Centrin staining. N=3 biological replicates. **D.** Quantification of FLAG+ cells with >10 cilia at additional timepoints (Day 14 and 21)

from 3 biological replicates. Mean and SD are shown. **E.** Representative images of GEMC1+dBRD9 cells used for scoring in Figure 5C. **F**. BioID-AP followed by western blotting at Day 3. I indicates Input and E indicates Eluate. I and E westerns run separately, but from same BioID-AP sample. Similar results were obtained in 3 independent experiments.

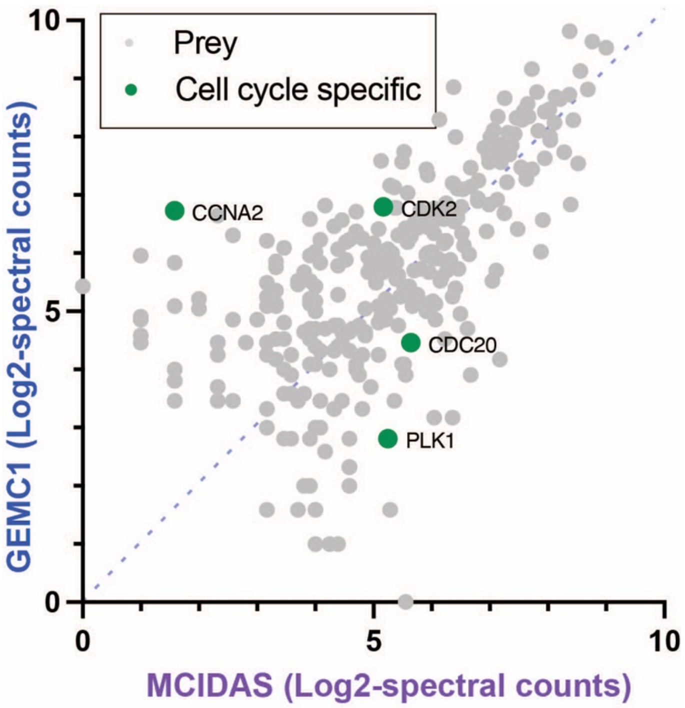

Supplementary Figure S7. Cell cycle specific proteins identified in BioID-MS. Scatterplot of data shown in Figure 2C highlighting proteins associated with specific cell cycle phases.
